# Rapid Diminution in the Level and Activity of DNA-Dependent Protein Kinase in Cancer Cells by a Reactive Nitro-Benzoxadiazole Compound

**DOI:** 10.3390/ijms17050703

**Published:** 2016-05-11

**Authors:** Viviane A. O. Silva, Florian Lafont, Houda Benhelli-Mokrani, Magali Le Breton, Philippe Hulin, Thomas Chabot, François Paris, Vehary Sakanyan, Fabrice Fleury

**Affiliations:** 1 UFIP CNRS UMR 6286, Mechanism and Regulation of DNA Repair Team, Faculté des Sciences et des Techniques, Université de Nantes, 2 rue de la Houssinière, 44322 Nantes, France; vivianeaos@gmail.com (V.A.O.S.); florian.lafont@etu.univ-nantes.fr (F.L.); houda.benhelli@univ-nantes.fr (H.B.-M.); magali.lebreton@univ-nantes.fr (M.L.B.); thomas.chabot1@etu.univ-nantes.fr (T.C.); 2 Plate-forme MicroPICell SFR Santé F. Bonamy-FED 4203/Inserm UMS016/CNRS UMS3556, 44007 Nantes, France; philippe.hulin@univ-nantes.fr; 3 UMR 892 Inserm-6299 CNRS, team 14, 44007 Nantes, France; francois.paris@inserm.fr; 4 IICiMed EA-1155, Faculté de Pharmacie, Faculté des Sciences et des Techniques, Université de Nantes, 2 rue de la Houssinière, 44322 Nantes, France; 5 ProtNeteomix, 29 rue de Provence, 44700 Orvault, France

**Keywords:** DNA-PKcs, DNA repair, hydrogen peroxide, SOD1, nitro-benzoxadiazole, chemiosensitization, protein targeting, prostate cancer

## Abstract

The expression and activity of DNA-dependent protein kinase (DNA-PK) is related to DNA repair status in the response of cells to exogenous and endogenous factors. Recent studies indicate that Epidermal Growth Factor Receptor (EGFR) is involved in modulating DNA-PK. It has been shown that a compound 4-nitro-7-[(1-oxidopyridin-2-yl)sulfanyl]-2,1,3-benzoxadiazole (NSC), bearing a nitro-benzoxadiazole (NBD) scaffold, enhances tyrosine phosphorylation of EGFR and triggers downstream signaling pathways. Here, we studied the behavior of DNA-PK and other DNA repair proteins in prostate cancer cells exposed to compound NSC. We showed that both the expression and activity of DNA-PKcs (catalytic subunit of DNA-PK) rapidly decreased upon exposure of cells to the compound. The decline in DNA-PKcs was associated with enhanced protein ubiquitination, indicating the activation of cellular proteasome. However, pretreatment of cells with thioglycerol abolished the action of compound NSC and restored the level of DNA-PKcs. Moreover, the decreased level of DNA-PKcs was associated with the production of intracellular hydrogen peroxide by stable dimeric forms of Cu/Zn SOD1 induced by NSC. Our findings indicate that reactive oxygen species and electrophilic intermediates, generated and accumulated during the redox transformation of NBD compounds, are primarily responsible for the rapid modulation of DNA-PKcs functions in cancer cells.

## 1. Introduction

DNA damage caused by different endogenous and exogenous factors triggers signaling and repair pathways, which are vital for the maintenance of genome integrity [1]. DNA double-strand breaks (DSBs) are considered the most cytotoxic DNA lesions; they result from endogenous events such as V(D)J recombination and the production of reactive oxygen species (ROS) during cellular metabolism, as well as from exogenous sources such as ionizing radiation and radiomimetic drugs [2]. The two main mechanisms by which cells repair DSBs are non-homologous end joining (NHEJ) and homologous recombination (HR). During NHEJ, the two broken ends of DNA are pieced together, sometimes after limited processing of these ends, resulting in quick error-prone repair [3]. The core components of NHEJ include DNA-dependent protein kinase (DNA-PK), which is composed of DNA-binding subunits (Ku70 and Ku86) and a catalytic subunit (DNA-PKcs), DNA ligase IV, the nuclease Artemis, and XRCC4 [4].

The 450-kDa DNA-PKcs is a key protein in the NHEJ pathway of DSB repair [2]. On the basis of sequence comparison, DNA-PKcs has been classified as a member of the phosphatidylinositol-3-kinase (PI-3-K)-related kinase (PIKK) super family of proteins [5], which also includes the ataxia telangiectasia mutated (ATM) and ATM-Rad3 related (ATR) proteins [6,7]. The role of DNA-PKcs in DNA-damage NHEJ and HR repair pathways, and its more recently discovered role in the regulation of the homeostasis of cell proliferation, have been extensively studied [8]. DNA-PKcs, along with ATM and ATR, is regarded more as a sensor of primary DNA damage than an inducible downstream effector of DNA damage signaling. The activation of DNA-PKcs may involve interactions with DNA and other proteins [9]. DNA-PKcs is abundantly expressed in almost all mammalian cells, is considered to be a predominantly nuclear protein, and appears to be present together with the Ku70/80 heterodimer in lipid rafts [10]. DNA-PKcs is subject to autophosphorylation and is involved in the phosphorylation of a range of protein substrates, including H2AX, p-53, Replication Protein A (RPA), and Artemis [4,11,12,13,14]. Recent findings indicate that the epidermal growth factor receptor (EGFR) is involved in modulating DNA double-strand break repair by NHEJ beyond modifying proliferation, survival, metastasis, and angiogenesis [15,16]. This modulation is especially important in tumors characterized by substantial over-expression of EGFR [17,18], including prostate cancer, in which the progression of the normal epithelium to an androgen-dependent tumor involves the activation of EGFR [19]. The efficiency of NHEJ is stimulated by a truncated EGFRvIII variant of the receptor, and reduced by EGFR-specific inhibitors in treated cells [20]. Experimental results have indicated that EGFR activation by various stimuli, such as ionizing radiation, ligand stimulation, heat shock, H_2_O_2_, and cisplatin, leads to the internalization of the receptor and its nuclear translocation, together with DNA-PK subunits [15]. The EGFR-mediated stimulation of DSB rejoining could be due to an increase in the nuclear content of DNA-PK subunits and hence an increase in the activity of the DNA-PK-dependent non-homologous end-joining (D-NHEJ) system [20]. Furthermore, it has been demonstrated that blocking EGFR-mediated signaling by cetuximab, an anti-EGFR monoclonal antibody, or by BIBX1382BS, a tyrosine kinase inhibitor of ErbB, results in a decrease in DNA-PK activity and sequestration of DNA-PK into the cytosol [21,22,23,24].

Recently, we have developed small molecule microarrays to screen chemical compound libraries with the goal of selecting molecules able to bind to the extracellular region of EGFR [25]. Among these small molecule binders, the compound NSC 228155 (referred to as NSC hereafter, Figure S1) carrying a nitro-benzoxadiazole (NBD) ring has been identified as an attractive non-peptide molecule that rapidly enhances tyrosine phosphorylation of EGFR and triggers downstream signaling pathways in cancer cells. However, the enhanced tyrosine phosphorylation of EGFR by NSC has been shown to be associated with the inhibition of protein phosphatase activity, suggesting that reactive oxygen species (ROS) contribute to activating the receptor [25]. 

In this study, we focus on the action of NSC on DNA damage repair proteins in prostate cancer cells. We show that exposure of cells to NSC leads to the rapid modulation of DNA-PKcs and other PIKK proteins. Our results reveal that the response of DNA repair proteins is primarily caused by reactive molecules generated during redox transformation of NSC in exposed cells. We also demonstrate that pretreatment of cancer cells with NSC sensitizes them to camptothecin-induced DNA breaks.

## 2. Results

### 2.1. NSC Treatment Rapidly Affects the Amount of DNA-PKcs Protein

**Figure 1:**
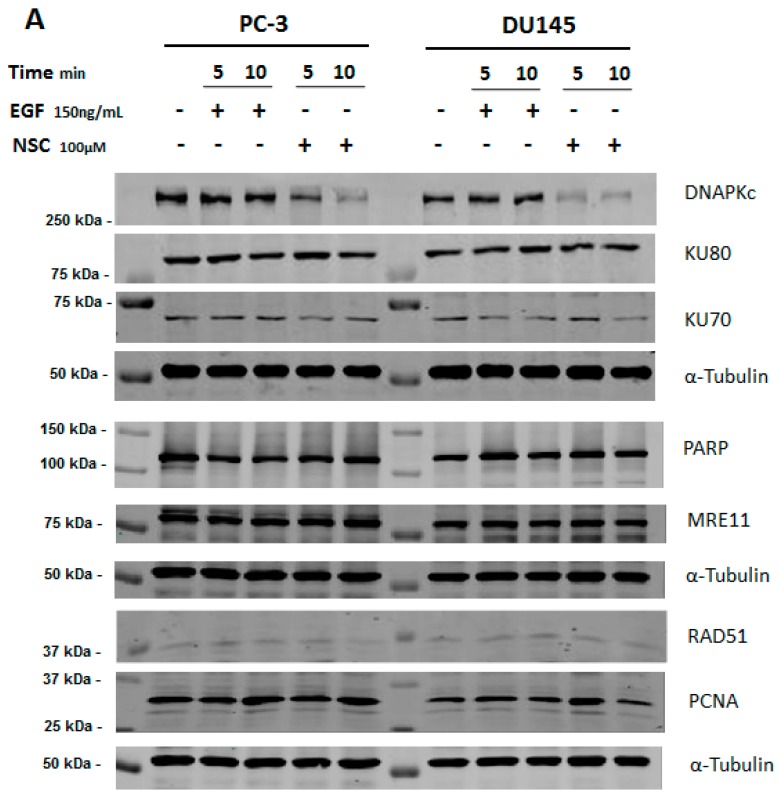

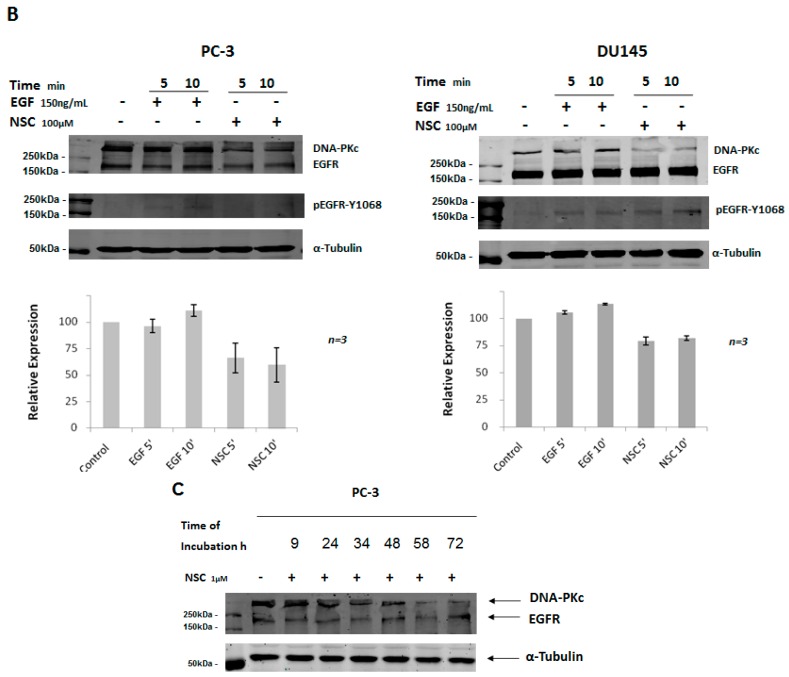
Relative level of DNA repair proteins in prostate cancer cells after exposure to compound NSC. (**A**) Levels of Rad51, PARP, MRE11, PCNA, EGFR, DNA-PKcs and KU DNA-binding subunits of DNA-PK were assessed in serum-starved PC-3 and DU145 cells, which were incubated with NSC (100 μM), or EGF (150 ng/mL), or vehicle (DMSO) for 5 and 10 min; (**B**) Quantitative analysis of the fluorescent signal intensity of protein bands shows that the decrease in the amount of DNA-PKcs is more pronounced in PC-3 than in DU145 cells briefly exposed to NSC; (**C**) Longer exposure of PC-3 cells to low concentrations of NSC (1 μM in this assay) also caused a gradual decrease in the DNA-PKcs expression level, but had almost no effect on the expression of EGFR in these conditions.

The amounts of major DNA repair proteins were assessed in prostate cancer cell lines PC-3 and DU145 treated by the small molecule NSC using Western immunoblotting. No difference was detected in the amounts of the DNA-binding subunits KU70 and KU80 of DNA-PK compared to the control and EGF treatment (Figure 1A). However, a clear decrease was found in the amount of the catalytic subunit of the protein, DNA-PKcs, in the same cells after exposure to 100 µM NSC for 5, and especially for 10 min. The relative amount of DNA-PKcs was lower in PC-3 than in DU145-treated cells, corresponding to a decrease of 40% and 20%, respectively (Figure 1B). None of the other DNA repair proteins tested, namely RAD51, PARP, PCNA, and MRE11, showed a significant difference in NSC-treated PC-3 and DU145 cells compared to untreated cells after 5 and 10 min of exposure (see Figure 1A). Meanwhile, the level of phosphorylated EGFR at Y1068 was enhanced in cells exposed to NSC or EGF (control), whereas the total amount of the receptor was not affected (Figure 1B).

In addition, the level of DNA-PKcs expression was assessed in PC-3 cells after exposure to NSC at lower concentrations and longer incubation times, up to 72 h. In contrast to EGFR, a gradual decrease in the DNA-PKcs protein level was observed (Figure 1C), indicating that the small molecule action on DNA-PKcs is dose-dependent and might have a cumulative effect on NHEJ repair efficiency and the viability of treated cells.

The rapid and selective decrease in the amount of DNA-PKcs in NSC-treated cells led us to assume that the protein might be subjected to degradation rather than the down-regulation of its expression under the conditions used. This assumption was first verified by RT-PCR analysis, which revealed that the amount of DNA-PKcs mRNA was not reduced in NSC-treated cells (Figure S2).

### 2.2. Subcellular Distribution of DNA-PKcs 

**Figure 2:**
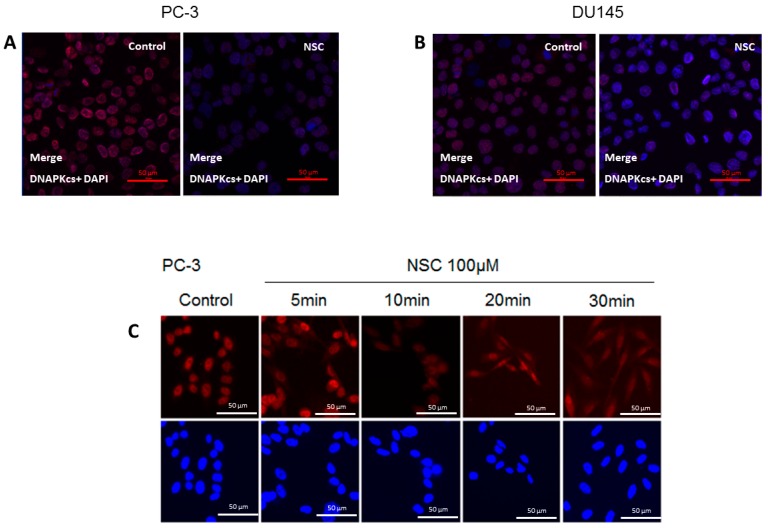
Intracellular localization of DNA-PKcs in prostate cells exposed to NSC. PC-3 (**A**) and DU145 (**B**) cells exposed to 100 μM NSC (NSC) or to vehicle (Control) for 10 min; (**C**) DNA-PKcs intracellular distribution in PC-3 was also analyzed after NSC treatment at 100 µM for 5, 10, 20 and 30 min. The treated and untreated cells were fixed and stained with anti-DNA-PKcs antibody (red color). The nuclei were stained with DAPI (blue color).

The subcellular distribution of DNA-PKcs in PC-3 and DU145 cells exposed to NSC was examined by immunostaining with an antibody specifically recognizing the catalytic subunit of the protein. Before NSC treatment, DNA-PKcs was mainly localized in the nucleus, which was stained with DAPI. After NSC incubation for 10 min, there was a visible decrease in DNA-PKcs protein levels in PC-3 and DU145 cells compared to non-treated control cells (Figure 2A,B). This result is consistent with the Western-blot analysis showing a weak fluorescence signal from DNA-PKcs in NSC-treated cells (Figure 1A). There was no apparent change in the subcellular distribution of DNA-PKcs under the measurement conditions used in Figure 2A,B. However, when a higher-resolution imaging was used, DNA-PKcs appeared to be distributed inside PC-3 cells shortly after exposure to NSC (Figure 2C). The fluorescent signal from DNA-PKcs had an essentially nuclear localization in control cells, whereas as little as a 5-min exposure to NSC resulted in the appearance of the protein-specific signal from the cytoplasm of many cells. This tendency became more clearly visible after a 10-min, 20-min and 30-min of exposure to NSC, indicating that DNA-PKcs was diffused in the cells.

Thus, a short exposure of cells to NSC results in a rapid decrease in the amount of nuclear DNA-PKcs, which is accompanied by delocalization of the protein into the cytoplasm of treated cells.

### 2.3. Exposure to NSC Alters the Activity of DNA-PKcs

Because of the low yield of phosphorylated forms of DNA-PKcs, the phosphorylation level of the protein was difficult to assess by Western blotting (Figure S3). Therefore, DNA-PKcs activity was evaluated indirectly by estimating the phosphorylation level of its substrate-protein RPA2. To address this, camptothecin (CPT), a selective topoisomerase I poison, was used as it promotes DNA-PK activation and subsequent RPA2 phosphorylation after treatment [26].

**Figure 3:**
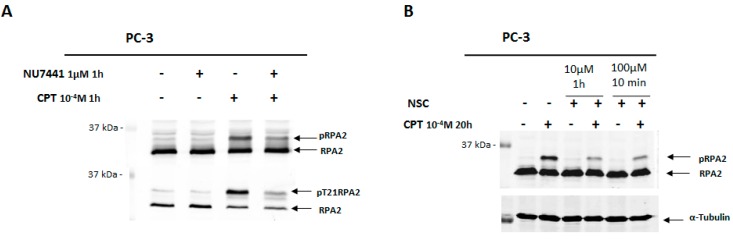
Assessment of DNA-PKcs activity through phosphorylation of a substrate protein RPA2 in PC-3 cells exposed to NSC. (**A**) PC-3 cells were incubated with 1 µM NU7441 (DNA-PKcs inhibitor) for 1 h, and then incubated with 100 µM CPT (topoisomerase I inhibitor) for 1 h. Phosphorylated forms of RPA were detected with anti-RPA2 and anti-pT21-RPA2; (**B**) After pre-incubation of serum-starved cells with 10^−4^ M CPT for 20 h and further incubation with 10 µM NSC for 1 h or 100 µM NSC for 10 min, the DNA-PKcs activity was analyzed by comparing the fluorescent intensity of phosphorylated RPA2 (hyperphosphorylation status) with anti-pT21-RPA2 antibody. α-Tubulin was used as an internal control.

As shown in Figure 3A, 1-h of treatment of PC-3 cells with 100 µM induced the phosphorylation of RPA2, which could be detected as bands with different migration velocities. These bands correspond to the intermediate and hyperphosphorylation status of RPA2 and the latter (the band with a lower migration) is directly related to DNA-PKcs activity [27]. In fact, pre-incubation with the inhibitor NU7441 strongly decreased this phosphorylated form of RPA2 indicating that DNA-PKcs activity was reduced in cells after CPT treatment (Figure 3A).

Next, PC-3 cells were pre-incubated with 0.1 mM CPT for 20 h, and then incubated with NSC at 10 µM for 1 h, or at 100 µM for 10 min. Western blotting analysis revealed that exposure to NSC remarkably decreased the phosphorylation of RPA2 in CPT pretreated cells in the conditions used (Figure 3B). These data indicate that NSC leads to rapid attenuation of the kinase activity of DNA-PK after CPT treatment in prostate cancer cells.

### 2.4. Exposure to NSC Promotes Protein Ubiquitination

**Figure 4:**
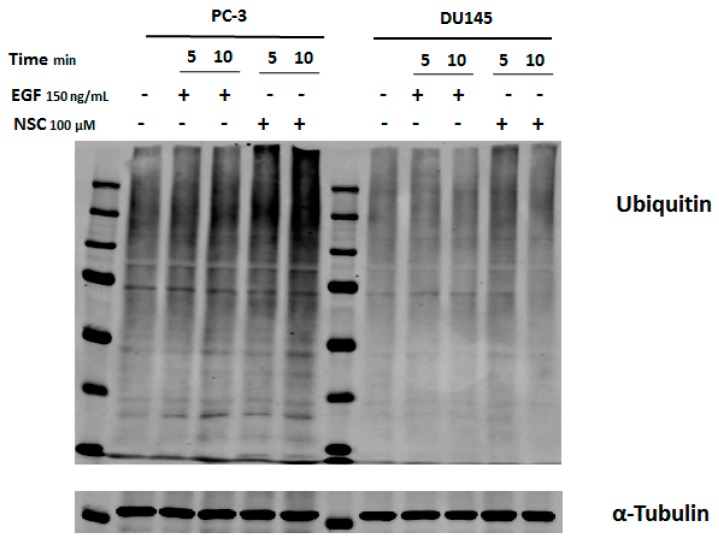
Assessment of protein ubiquitination in cancer cells exposed to EGF and NSC. Serum-starved PC-3 and DU145 cells were exposed to NSC (100 μM), or to EGF (150 ng/mL) for 5 or 10 min. Cell lysates were analyzed with anti-ubiquitin antibody recognizing ubiquitinated sites in proteins.

Previously, we showed that tyrosine phosphorylation (Tyr774) of the adaptor protein c-CBL located downstream of EGFR in the ubiquitination cascade was slightly increased in breast cancer cells shortly after exposure to compound NSC [25]. The proteasome-mediated proteolytic pathway is characterized by polyubiquitin chain formation on target proteins [27]. To find out whether the decrease in the amount of DNA-PKcs in cells after exposure to NSC is related to protein degradation, total protein ubiquitination was compared in non-treated and treated cells. Cell lysates of the samples obtained after exposure to NSC or EGF (control) were analyzed by Western blotting using an anti-ubiquitin antibody. It was observed that a 5-min and 10-min exposure to NSC in PC-3 or DU145 cells increased the fluorescence intensity of protein bands, especially for the higher molecular weight proteins, compared to control cells and those having been exposed to the peptide ligand EGF (Figure 4). The ubiquitination level was more pronounced in PC-3 cells than in DU145 cells.

These results show that NSC exposure rapidly promotes protein ubiquitination, which can activate the cellular proteasome and further degradation of DNA-PKcs.

### 2.5. NSC Inhibits DNA-PK Activity Differently from a Proteasome Inhibitor

**Figure 5:**
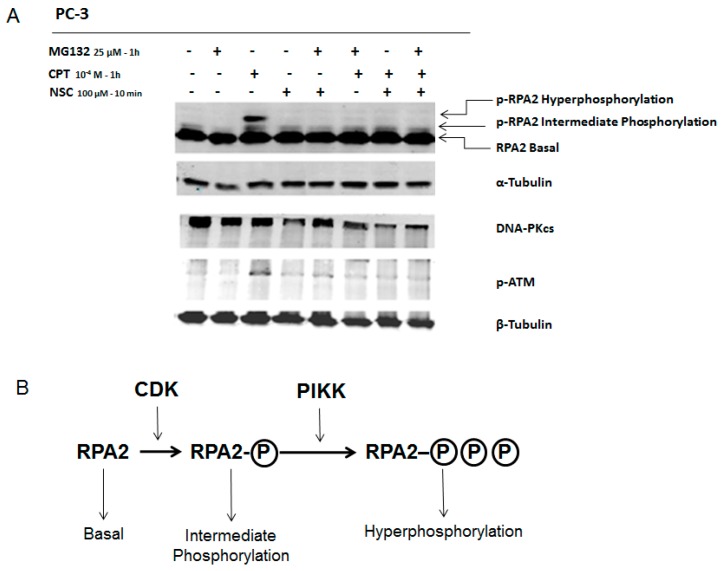
Response of DNA-PKcs and ATM to exposure to NSC and proteasome inhibitor MG-132 in CPT-treated PC-3 cells. (**A**) Serum-starved cells were incubated with 10^−4^ M CPT alone for 20 h and further exposed to NSC (100 µM for 10 min) or MG132 (25 µM for 1 h) alone or successively to the two compounds. Two phosphorylated forms of RPA2 are indicated; hyperphosphorylated and intermediate phosphorylated, which present high and middle bands, respectively. α-Tubulin was used as an internal control; (**B**) Schematic presentation of RPA2 phosphorylation routes through Cyclin-dependent kinases (CDKs) and PIKKs. CDKs contribute to RPA2 phosphorylation in a cell cycle-specific manner. Hyperphosphorylation of RPA2 is dependent on PIKKs, including ATM and DNA-PKcs.

To better understand the mode of modulation of DNA-PKcs activity by NSC, protein degradation was inhibited by pre-incubation of cells with a specific proteasome inhibitor. Previously, it was shown that the compound MG-132 suppresses CPT-induced DNA-PKcs autophosphorylation, which is specifically regulated by the proteasome in response to CPT [28]. PC-3 cells were incubated with one of these compounds, *i.e.*, CPT, NSC, or MG-132, or pretreated with CPT and then exposed to NSC or MG-132, or treated consecutively with NSC and then MG-132 (Figure 5). CPT treatment alone enhanced the phosphorylation of RPA2, as observed for the p-RPA2 intermediate and hyperphosphorylated forms, and increased ATM phosphorylation, but did not affect the amount of DNA-PKcs synthesized in the cells (Figure 5A, lane 3). As expected, incubation of cells with MG-132 strongly inhibited RPA2 phosphorylation in the CPT-pretreated culture (Figure 5A, lane 6) and a similar inhibition was detected after NSC exposure (Figure 5A, lane 7). Moreover, exposure to MG-132 or NSC decreased the phosphorylation level of ATM in CPT-pretreated cells. However, as judged by comparing the signal intensity recorded from the DNA-PKcs band in different samples, MG-132 pretreatment prevented the NSC-promoted decline in this key repair protein in the cells (Figure 5A, lane 5). The suppression of the action of NSC by the proteasome inhibitor is consistent with the assumption that NSC results in the degradation of DNA-PKcs through proteasome activation in cells. CPT-associated NSC treatment also decreased the ATM phosphorylation level (Figure 5A, lane 7), thus confirming the effect of NSC on the PIKK protein family.

### 2.6. Thioglycerol Prevents the Action of NSC on DNA-PKcs

When this study was nearing completion, we revealed that the enhanced phosphorylation of EGFR by NBD compounds is a result of the generation and action of hydrogen peroxide on the receptor in breast cancer cells [29]. In particular, it was shown that lipophilic NBD compounds rapidly bind to intracellular Cu/Zn superoxide dismutase 1 (SOD1) and thereby induce its dimerization and the production of H_2_O_2_ in cells. Pre-incubation of cells with thioglycerol prevented SOD1 dimerization, and the inactive enzyme was no longer able to convert the superoxide ion into H_2_O_2_.

**Figure 6:**
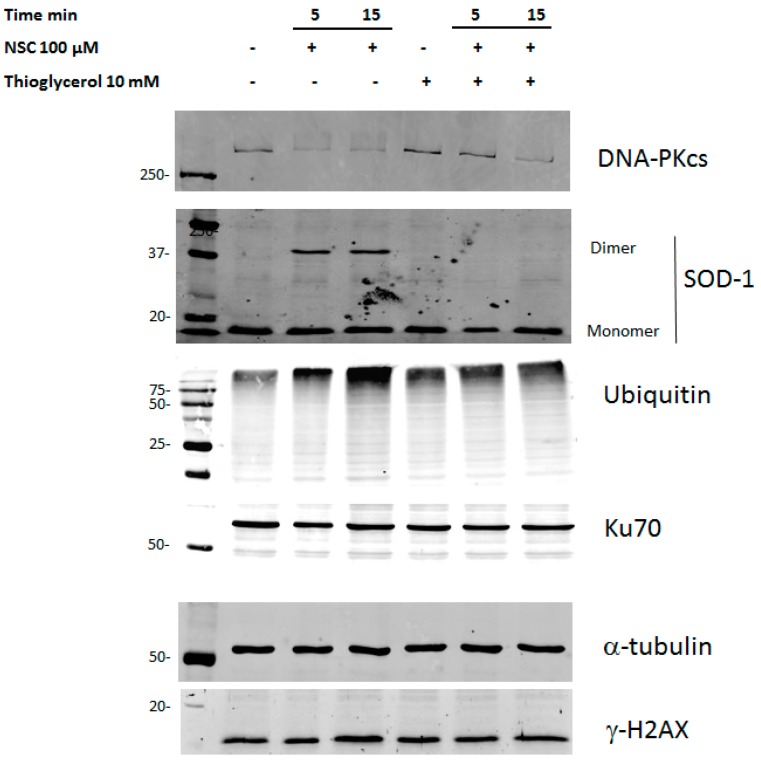
Pre-incubation with thioglycerol restores the level of DNA-PKcs by preventing dimerization of SOD1 in cancer cells exposed to NSC. PC-3 cells were pre-incubated with 10 mM thioglycerol for 15 min then incubated with 100 µM NSC for 5 or 10 min. Proteins were analyzed by Western blotting with the corresponding antibodies to detect DNA-PKcs, anti-SOD-1, ubiquitinated targets and γH2AX. Ku70 was used as a negative control (see above) and α-tubulin as a loading control.

To ascertain whether ROS are involved in the modulation of DNA-PKcs expression, the PC-3 culture was first pretreated with thioglycerol for 30 min and then exposed to NSC for 5 or 15 min. Western-blot analysis showed that pre-incubation with the antioxidant almost completely restored the amount of DNA-PKcs after 5-min of exposure to NSC, which was still clearly visible after 15-min of exposure (Figure 6). It is noteworthy that the preventative effect of thioglycerol was accompanied by the complete disappearance of SOD1 dimers. Besides, the decrease in the amount of DNA-PKcs after pretreatment with thioglycerol was also accompanied by a decrease in the proteasome-specific bands after 15-min of exposure to NSC that was not yet visible after 5-min of exposure. Meanwhile, the amounts of the proteins Ku70 and gamma-H2AX remained unchanged in the cells pre-incubated with thioglycerol, regardless of exposure to NSC.

To confirm the role of SOD1 in the diminution of DNA-PKcs expression after exposure to NSC, we addressed MDA MB468 breast cancer cells, in which SOD1 was the knockdown with siRNA [29]. Western blotting revealed the presence of two bands corresponding to DNA-PKcs monomer and dimer forms (or large complexes formed with other partners) in cells transfected with SOD1 siRNA or scrambled siRNA (Figure S4). The amount of DNA-PKcs monomer forms prevails over dimers in cells treated with vehicle, whereas the amount of DNA-PKcs dimers prevails over monomers after exposure to NSC. However, the amount of both forms of DNA-PKcs was remarkably lower in cells transfected with scrambled siRNA than with SOD1 siRNA after exposure to NSC. The diminution in the level of DNA-PKcs in cells exposed to NSC correlated with the dimerization rate of SOD1 (see Figure S4). Thus, these data indicate that the decrease in the level of DNA-PKcs occurs via rapid dimerization of SOD1 by NSC. SOD1 is required to produce hydrogen peroxide, which is the reactive oxygen species that modulates the expression level of DNA-PKcs.

### 2.7. NSC Can Sensitize Cancer Cell Lines to Camptothecin-Mediated DNA Damage

DNA-PKcs has been widely used as a target to sensitize cancer cells to small molecules exhibiting anticancer potency [30,31,32]. Given that exposure to NSC attenuated the kinase activity of DNA-PK in PC-3 cells pretreated with CPT (see above), it was tempting to improve the sensitivity of prostate cancer cells to the topoisomerase I poison by pre-incubation of cells with NSC.

**Figure 7:**
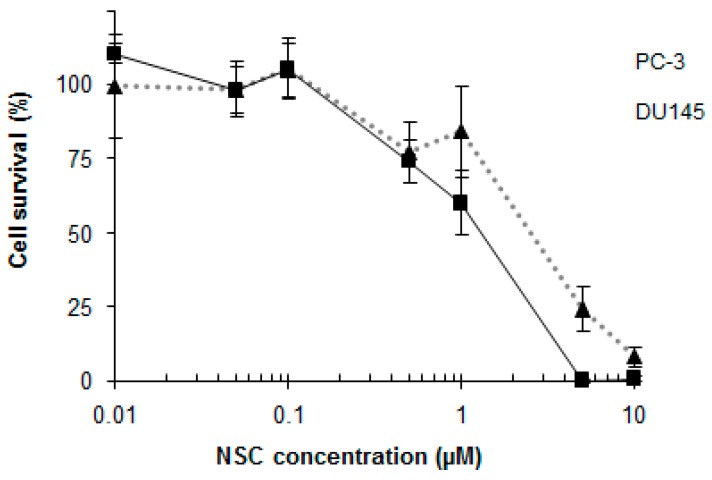
PC-3 and DU145 cell survival is dose-dependent in NSC-treated cells. PC-3 cells (dashed gray line) and DU145 cells (black line) were incubated with different concentrations of NSC for 72 h and cellular viability was measured by the MTT method. The 50% inhibitory concentration (IC_50_) of NSC was calculated by nonlinear regression analysis using GraphPad Prism software. The bars represent the mean ± SD of 3 independent experiments.

The viability of cancer cells was determined by incubating PC-3 and DU145 cells with different concentrations of NSC for 72 h. This resulted in a significant reduction in the survival of cells with IC_50_ values of 2.65 μM and 1.25 μM for PC-3 and DU145 cells, respectively (Figure 7). PC-3 cells were more resistant to NSC exposure and were characterized by a higher amount of DNA-PK protein in comparison to DU145 cells (see Figure 1A,B).

**Figure 8:**
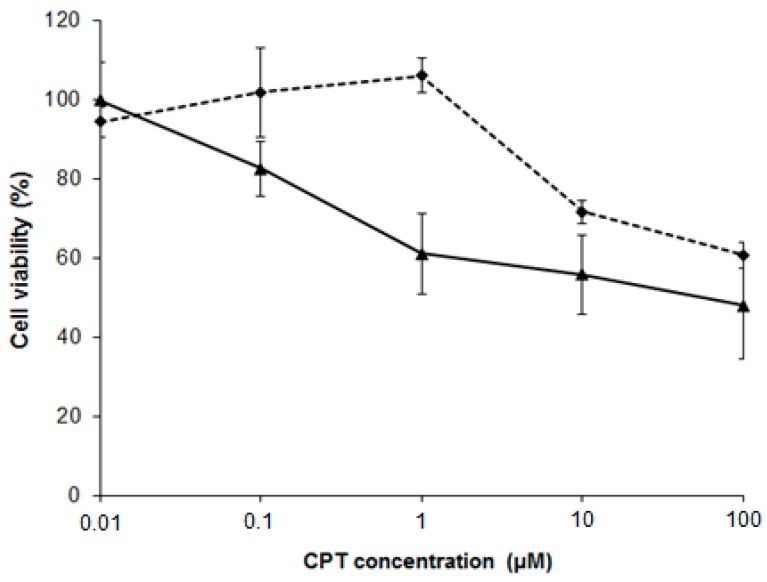
NSC pretreatment improves the cytotoxic effect of camptothecin on PC-3 cells. Cells were pretreated with 100 µM NSC for 10 min (solid line) or untreated (dashed line), washed, and further exposed to camptothecin at different concentrations for 48 h.

Next, PC-3 cells were treated with 100 µM NSC for 10 min and then incubated with CPT for 48 h. Cell viability was assessed by the MTT method. As shown in Figure 8, CPT treatment after exposure to NSC decreased the viability of PC-3 cells in comparison with CPT treatment alone. This chemosensitizing effect was especially pronounced at 1 µM CPT. The ability of NSC to increase the cytotoxicity of CPT in PC-3 cells leads us to suggest that this small compound acts through the modulation of DNA-PKcs functions and levels, apparently by altering the NHEJ repair system in cells sensitized by camptothecin. However, the sensitization of PC-3 cells by NSC could be related with targeting other vital proteins.

## 3. Discussion

Recently, we have described a new class of nitro-benzoxadiazole derivatives, which activate EGFR and thereby trigger downstream signaling circuits in the cytoplasm and apparently in the nucleus of breast cancer cells [25]. Here, we show that exposure of prostate cancer cell lines DU145, and especially PC-3, to the compound NSC rapidly (within 5–10 min) reduced the amount and activity of DNA-PKcs protein. Exposure to NSC sensitized prostate cancer cells to camptothecin-mediated DNA damage in a time-dependent manner. The decrease in the amount of DNA-PK protein is not related to the delocalization of DNA-PK subunits into the nucleus, unlike in EGF-induced cells [20]. In contrast, this decrease is accompanied by a nuclear delocalization of DNA-PKcs into the cytoplasm, which probably promotes rapid protein ubiquitination in cells exposed to NSC compared to a cognate ligand EGF. Therefore, ubiquitin-mediated degradation of DNA-PKcs, the key partner of NHEJ, might affect the functions of the protein required for interactions with DNA and other repair proteins.

DNA-PKcs is involved in the phosphorylation of a range of substrates, including H2AX, p-53, RPA, and Artemis [4]. DNA-PKcs undergoes autophosphorylation within two distinct regions known as the ABCDE (T2609, S2612, T2620, S2624, T2638, and T2647) and PQR (S2023, S2029, S2041, S2051, S2053, and S2056) clusters [11,12,13,14]. Mutations of these phosphorylation sites result in ablated DSBs repair and radiosensitivity *in vitro* and *in vivo*, suggesting that the autophosphorylation of DNA-PKcs is critical for NHEJ efficiency. It therefore seems that other kinases are involved in its phosphorylation, the main one being ATM, a member of the PIKK protein super family [33]. In addition, ATR is responsible for the phosphorylation of T2609 and T2647 but phosphorylation of these residues, in contrast to that of serine 2056, is not an appropriate marker for the activation of DNA-PKcs [33,34,35,36]. It is worth noting that all three proteins, *i.e.*, ATM, ATR, and DNA-PK, are considered sensors of primary DNA damage rather than inducible downstream effectors of DNA damage signaling [6,7].

In this study, a remarkable diminution in RPA2 phosphorylation was observed upon NSC treatment combined with CPT, indicating that the nitro-benzoxadiazole scaffold attenuates DNA-PKcs kinase activity. Moreover, ATM phosphorylation (S1981) decreased upon NSC exposure in response to DNA damage. Because of the low yield of DNA-PKcs phosphorylated protein forms, DNA-PKcs activity was assessed by the phosphorylation rate of its substrate, RPA2, when cells were induced by a selective topoisomerase I poison, CPT [26]. It has been demonstrated that RPA2 can be phosphorylated in two ways: by a cyclin-dependent kinase (CDK) and by PIKKs (Figure 5B). CDKs contribute to RPA2 phosphorylation in a cell cycle-specific manner [28]. In addition, several PIKKs, including ATM and DNA-PK, are involved in the hyperphosphorylation of RPA2 [37,38]. The analysis of the phosphorylated bands of RPA2 indicates that MG-132 suppresses RPA2 phosphorylation by PIKKs (absence of a hyperphosphorylated RPA2 band in Figure 5A, lane 6, and explained in Figure 5B), but not by CDK in CPT-treated cells, in agreement with the observations of other authors [28]. This is also supported by findings that MG-132 pretreatment prevents RPA2 phosphorylation by CDK after further exposure to NSC in CPT-incubated cells; on the contrary, when MG-132 is added to CPT-incubated cells after NSC exposure, RPA2 phosphorylation by CDK is suppressed (Figure 5C). These results suggest that NSC modulates CDK activity in addition to DNA-PKcs by the proteasome machinery in cancer cells.

We have recently shown that lipophilic NBD compounds rapidly move through the cytoplasm membrane and bind to Cu/Zn SOD1 leading to dimerization of the protein [29]. Stable dimers of SOD1 are active and produce H_2_O_2_ that, in the absence of adequate modulation of catalase and peroxidase activities, is accumulated within exposed cells (*ibid*). Note that hydrogen peroxide promotes sulfenylation of a cysteine in the catalytic site of the protein tyrosine phosphatase 1B (PTP-1B), leading to the inactivation ofi the enzyme [39], and also promotes the sulfenylation of a cysteine in the catalytic site of EGFR leading to the activation of the enzyme [40]; hence, both enzymes determine the phosphorylation status of the receptor in cells. Therefore, the enhanced phosphorylation of EGFR is a result of hydrogen peroxide action on the catalytic site of EGFR and that of PTP-1B in cells exposed to NBD compounds [29].

Similarly, our data highlight that hydrogen peroxide is generated and accumulates during redox transformation of the NBD compounds [29,41], and is involved in the decline in the level and activity of DNA-PKcs in prostate cancer cells. In fact, given that thioglycerol prevents SOD1 dimerization by NSC, the inactive enzyme cannot convert superoxide ions into H_2_O_2_ and, therefore the amount of DNA-PKcs is almost restored in cells. This data is supported by SOD1 RNA interference in breast cancer cells. Thus, we conclude that rapid modulation of this DNA-repair protein by NSC is primarily related to the action of reactive H_2_O_2_, without involvement of EGFR functions.

Several scenarios can be suggested to explain the decrease in DNA-PKcs level in prostate cancer cells due to the action of H_2_O_2_, which is rapidly accumulated within cells exposed to NSC. Firstly, large quantities of intracellular H_2_O_2_ could immediately and directly target and degrade DNA-PKcs; Secondly, hydrogen peroxide stimulates protein degradation by up-regulation of the ubiquitin system [42]. In particular, it causes down-regulation of DNA-PKcs, which involves trapping the DNA-topoisomerase II complex and the induction of DNA double-strand breaks in salvicine-treated cells [43]. In this context, the cellular proteasome, which is activated by NSC, could essentially contribute to the degradation of DNA-PKcs and other DNA repair protein targets; Thirdly, the reduction of the nitro group in NBD compounds during redox cycling results in the formation of two types of reactive molecules, *i.e.*, ROS and electrophilic NBD intermediates [29,41].

Therefore, the decrease in the level of DNA-PKcs could be caused by electrophilic intermediates of NSC during redox transformations. The binding of electrophiles to the protein-protein interaction interface of DNA-PKcs could lead to the dimerization of the protein or the formation of a protein complex with other partners. Such a large and stable protein complex is degraded as could be suggested from observations in cells transfected with scrambled siRNA. Lastly, a reduction in the amount of DNA-PKcs might be caused by other factors that still need to be identified. In any event, the function of DNA-PKcs, one of the key players in DNA repair, would be impaired, along with impaired functions of SOD1 and EGFR, which must lead to aberrant signaling and aggravate oxidative and electrophilic stress in cells exposed to NBD compounds, and probably to some reactive environmental pollutants.

Due to its broad protective role, alterations in DNA-PK activity correlate with a variation in cell survival after radio- and chemotherapy, making this protein of great interest as a promising target for drug development [15]. Given that cell exposure to NSC affects proteins important in DNA damage signaling, it is plausible that a DNA repair defect may contribute to enhancing the cytotoxicity of damaging agents like topoisomerase I poisons. In this context, we have shown that exposure to NSC sensitizes PC-3 cells to CPT treatment. It is known that the activation of DNA-PK by CPT is strictly S-phase specific, suggesting that DNA-PK might promote NHEJ repair of CPT-induced damage in the S phase. Although the overall contribution of NHEJ to genomic stability in the S phase is still not clear, several reports have shown that DNA-PKcs-deficient cells are sensitive to UV and CPT [26,44], implying that DNA-PK might be required for stalled fork repair through HR or checkpoint activation, which is necessary for cell survival. In addition to its role in NHEJ repair, DNA-PKcs is also involved in the oxidative clustered DNA lesion repair [45] that could explain the ROS-mediated effect of NSC.

Further studying the action of reactive NBD compounds on DNA repair and related functions could help in elucidation of the complexity of events involved in the sensitization of cancer cells to anticancer agents.

## 4. Experimental Section

### 4.1. Chemicals and Antibodies

NSC 228155 (NSC) was obtained from the Drug Synthesis and Chemistry Branch of NCI (Figure S1). Camptothecin (CPT), NU7441, MG132 and thioglycerol were purchased from Sigma (St Louis, MO, USA). The anti-ATM and anti-RPA2 antibodies were purchased from Thermo Fisher Scientific (Waltham, MA, USA). The anti-phospho(T21)RPA and anti-phospho(S2056)DNA-PKcs antibodies were purchased from Abcam (Cambridge, UK); all others were purchased from Cell Signaling Technology (Danvers, MA, USA).

### 4.2. Cell Culture

The prostate cancer cell lines, PC-3 and DU145, were grown in RPMI 1640 medium (Life Technologies, Carlsbad, CA, USA) containing 10% fetal bovine serum, 100 U/mL penicillin and 100 µg/mL streptomycin under a humidified atmosphere containing 5% CO_2_ at 37 °C. If necessary, PC-3 and DU145 cells (1.0–1.2 × 10^6^/mL) were serum-starved for 24 h, then seeded into 60-mm culture dishes and treated with NSC alone or MG132 or camptothecin for the desired periods as described below.

### 4.3. Cell Viability Assay and Chemical Treatments

Cell viability was assessed by the MTT assay (Sigma) following the manufacturer’s instructions. Briefly, cells were seeded at 5 × 10^3^/well in a 96-well plate for 18 h, and then incubated in serum-starved RPMI 1640 medium for 24 h. Cell cultures were then treated with increasing concentrations of NSC for 72 h. MTT was added to each well for 2 h, before being aspirated and replaced with DMSO. The absorbance was recorded at 560 nm with a spectrophotometer system (Tecan, Männedorf, Switzerland). Results were analyzed using Microsoft Excel by standardizing treated groups to the untreated control. The IC_50_ was determined as the compound concentration leading to 50% growth inhibition.

Cells were also treated with 0.1 mM camptothecin (CPT) for 2 h at 37 °C or 10 μM MG132 for 1 h at 37 °C and then exposed to NSC as described in the corresponding sections. The ROS effect was investigated on cells pre-incubated with 10 mM thioglycerol for 15 min before exposure to 100 µM NSC for 5 or 15 min.

### 4.4. Western Blotting 

Cells were washed twice with ice-cold PBS and then lysed in lysis buffer (NP-40) on ice for 30 min. The soluble fraction of proteins was collected by centrifugation at 13,000× *g* for 15 min at 4 °C. The protein concentration was determined with the Bicinchoninic acid protein assay kit (BioRad, Hercules, CA, USA) and samples were either used immediately for assays or stored at −80 °C.

Equal amounts of cellular lysate proteins (40 µg of total proteins) were separated by electrophoresis on 10% SDS-polyacrylamide gels and transferred to nitrocellulose membranes (GE Healthcare, Little Chalfont, UK). Membranes were blocked for 1 h in 0.1% TBS-Tween 20 containing 2.5% BSA and then incubated with mouse monoclonal antibodies (anti-DNA-PKcs, anti-RPA2, anti-tubulin) or rabbit antibodies (anti-pATM, anti-ubiquitin). Protein bands were resolved by fluorescence with anti-mouse Alexa-Fluor680 or anti-rabbit Alexa-Fluor680 secondary antibodies (Life Technologies-Invitrogen). The signal intensity (pixel·mm^−2^) of protein bands was quantified using Odyssey software version 1.1 (Li-COR, Biosciences, Lincoln, NE, USA). α-Tubulin was used as a loading control.

### 4.5. Immunofluorescence Microscopy

Cells were grown on coverslips (Life Technologies) at a density of 5 × 10^3^ cells per well in medium (RPMI) supplemented with 10% fetal bovine serum (Gibco-Invitrogen) as described above, then serum-starved for 24 h and treated with NSC for 10 min. Cells were washed once in PBS for 5 min and fixed with 4% paraformaldehyde in 0.01 M phosphate-buffered saline (PBS), pH 7.4 for 10 min. After three 5-min washes in PBS, cells were permeabilized with PBS containing 0.1% Triton X-100 for 30 min, washed three times in PBS for 5 min, and then blocked in 2% BSA in PBS at room temperature for 25 min. The fixed cells were incubated overnight in the same buffer containing 1:100 diluted anti-DNA-PKcs antibody at 4 °C. After three 5-min washes in PBS, slides were incubated in a 1:400 TRITC anti-mouse fluorescent secondary antibody solution (Jackson, Lansing, MI, USA) for 1 h at room temperature in the dark. The slides were washed three times in PBS, counterstained and mounted with ProLong Antifade with DAPI (4’,6-diamidino-2-phenylindole) (Life Technologies) and coverslips were applied. The slides were viewed with a confocal microscope (Nikon A1RSi, Minato-ku, Tokyo, Japan) and epifluorescence microscope (Nikon Eclipse E800). The images were recorded with NIS Element software (Version 3.6, Nikon, Tokyo, Japan) and processed with the software ImageJ (NIH, Bethesda, MD, USA). The specificity of the antibody staining was confirmed by incubating the adjacent sections in the absence of the primary antibody.

### 4.6. Statistical Analysis

The results represent the average and standard deviation of three independent experiments. Data are presented as means ± standard error of mean. Differences between the control and the treated cells were assessed with the paired Student’s *t*-test, and *p* < 0.05 was considered statistically significant. Data analysis was performed with Microsoft Excel.

## 5. Conclusions

Our results indicate that a reactive NBD compound rapidly and drastically reduces the level and activity of DNA-PKcs and, alters the DNA repair system in cancer cells. The mechanism of action relies on the generation of ROS and electrophilic species leading to the activation of protein degradation. Notably, that NBD compound sensitizes prostate cancer cells to camptothecin. Therefore, developing NBD derivatives that selectively target DNA-PKcs might be a promising way to sensitize cancer cells in order to improve the efficiency of anticancer agents from a therapeutic perspective.

## Supplementary Materials

Supplementary materials can be found at http://www.mdpi.com/1422-0067/17/5/703/s1.

## Abbreviations

ATM	Ataxia Telangiectasia Mutated
ATR	ATM-Rad3 Related
CDKs	Cyclin-dependent kinases
CPT	Camptothecin
DNA-PKcs	DNA-dependent Protein Kinase catalytic subunit
DSBs	DNA double-strand breaks
EGFR	Epidermal Growth Factor Receptor
HR	Homologous Recombination
NBD	Nitro-BenzoxaDiazole
NHEJ	Non-Homologous End Joining
NSC	4-Nitro-7-[(1-oxidopyridin-2-yl)sulfanyl]-2,1,3-benzoxadiazole
PIKK	Phosphatidyl inositol 3′ kinase-related kinases
PTP-1B	Protein Tyrosine Phosphatase 1B
RPA	Replication Protein A
ROS	Reactive Oxygen Species
SOD1	Superoxide Dismutase 1
