# Supplementary material for: Rapid Diminution in the Level and Activity of DNA-Dependent Protein Kinase in Cancer Cells by a Reactive Nitro-Benzoxadiazole Compound
Source: Int J Mol Sci. 2016 May 11;17(5):703. doi: 10.3390/ijms17050703 (PMC4881526; doi:10.3390/ijms17050703)
Supplement: Supplementary file 1 [file ijms-17-00703-s001.pdf]

Supplementary Materials: Rapid Diminution in the Level and Activity of DNA-Dependent Protein Kinase in Cancer Cells by a Reactive Nitro-Benzoxadiazole Compound

Viviane A. O. Silva, Florian Lafont, Houda Benhelli-Mokrani, Magali LeBreton, Philippe Hulin, Thomas Chabot, François Paris, Vehary Sakanyan and Fabrice Fleury

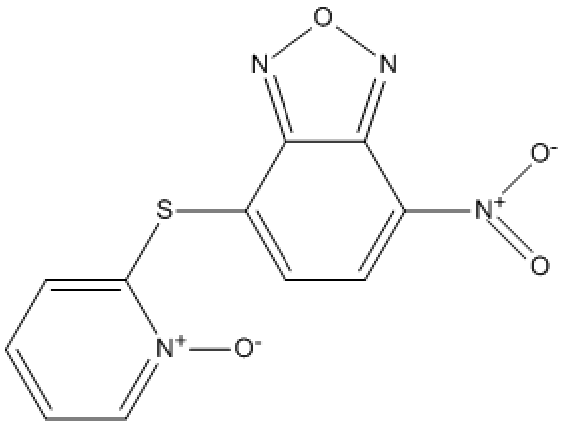

Figure S1. Chemical structure of compound NSC 228155. 4-nitro-7-[(1-oxidopyridin-2-yl)sulfanyl]-2,1,3-benzoxadiazole (described as NSC in the manuscript).

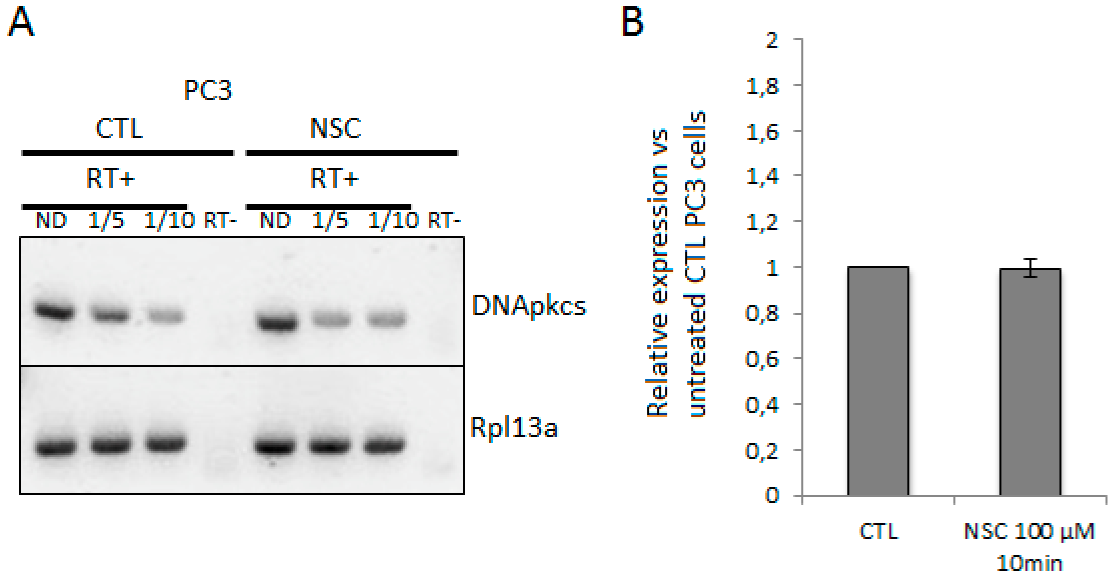

Figure S2. A decrease in the level of DNA-PKcs is not related to reduced gene expression in cells exposed to NSC. DNA-PKcs RNA expression was analyzed in PC-3 cells treated with 100 µM NSC for 10 min by RT-PCR (**A**) and quantified by qRT-PCR (**B**). The RNA level ratio in untreated PC-3 control cells was normalized to 1 and the average DNA-PKcs ratio in NSC-treated cells was expressed relative to the PC-3 control cells. Data are expressed as mean ± SD.

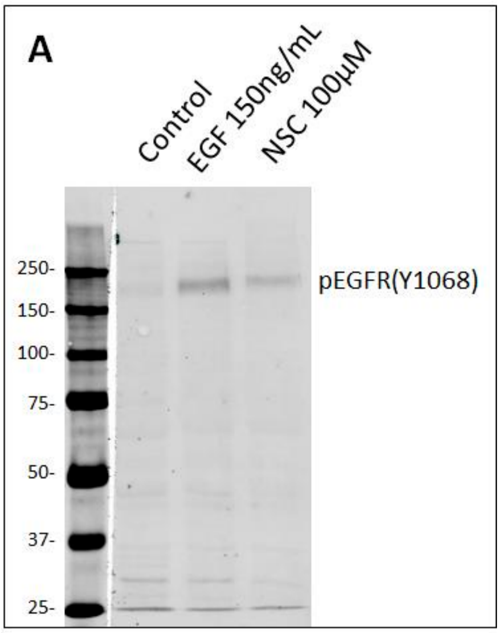

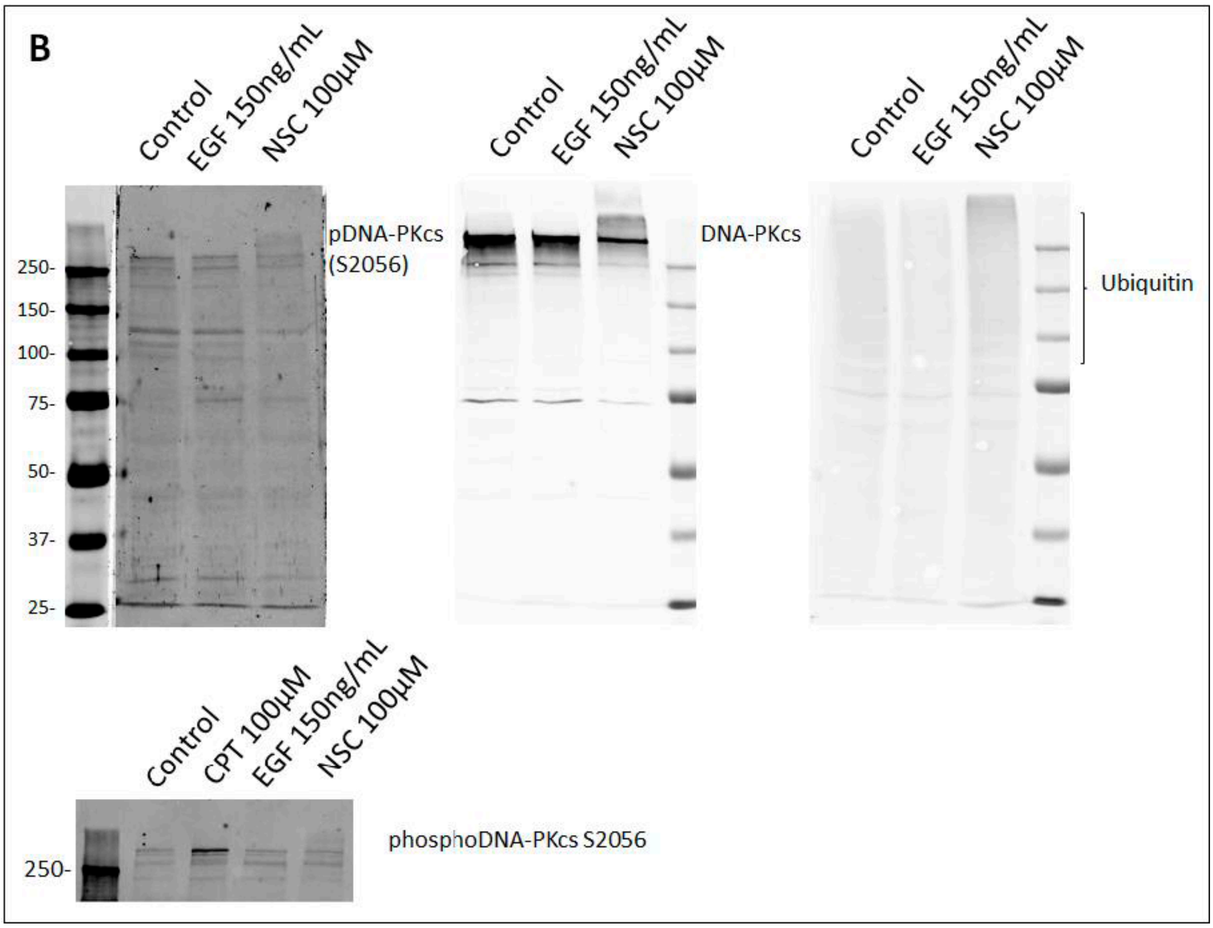

Figure S3. PC-3 cells treated with 150 ng/mL EGF or 100 µM NSC for 10 min. (**A**) Phosphorylation of EGFR is induced by EGF and NSC. NSC and EGF promote the phosphorylation of EGFR at Y1068; (**B**) The signal reduction of DNA-PKcs by NSC (middle image) is associated with an increased ubiquitination of some high molecular mass proteins (right image). The phosphorylation at S2056 is not clearly visible in EGF or NSC-treated cells (left image) in contrast to CPT- treatment (bottom image, blocking in 5% milk-TBST).

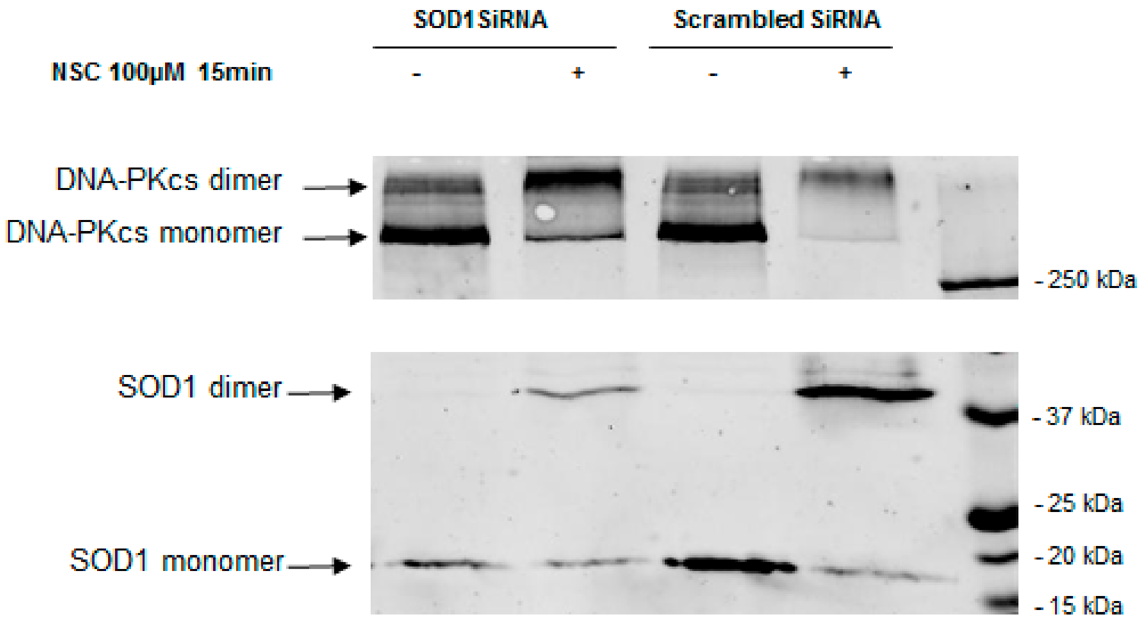

Figure S4. Effects of SOD1 siRNA interference on DNA-PKcs expression in breast cancer cells exposed to NSC. MDA-MB468 cells were transfected with SOD1 siRNA or scrambled siRNA and exposed to 100 µM NSC for 15 min [25]. 4 µg of total protein was loaded in each lane.
